# Epidemiological differences between the sexes in adolescent patients with lumbar spondylolysis: a single-institution experience in Japan

**DOI:** 10.1186/s12891-023-06679-1

**Published:** 2023-07-08

**Authors:** Reo Asai, Masaki Tatsumura, Hisanori Gamada, Shun Okuwaki, Fumihiko Eto, Katsuya Nagashima, Yousuke Takeuchi, Toru Funayama, Takeo Mammoto, Atsushi Hirano, Masashi Yamazaki

**Affiliations:** 1 Department of Medical Education and Training, Tsukuba Medical Center Hospital, 1-3-1, Amakubo, Tsukuba, Ibaraki 305-8558 Japan; 2 Department of Orthopaedic Surgery and Sports Medicine, Tsukuba University Hospital Mito Clinical Education and Training Center, Mito Kyodo General Hospital, 3-2-7, Miya-machi, Mito, Ibaraki 310-0015 Japan; 3 Department of Orthopaedic Surgery, Institute of Medicine, University of Tsukuba, 1-1-1, Tennodai, Tsukuba, 305-8575 Japan

**Keywords:** Lumbar spondylolysis, Sex difference, Adolescent, Spina bifida occulta, Bone marrow edema, L5

## Abstract

**Background:**

Lumbar spondylolysis, a common identifiable cause of low back pain in young athletes, reportedly has a higher incidence rate in males. However, the reason for its higher incidence in males is not clear. This study aimed to investigate the epidemiological differences between the sexes in adolescent patients with lumbar spondylolysis.

**Methods:**

A retrospective study was conducted in 197 males and 64 females diagnosed with lumbar spondylolysis. These patients visited our institution from April 2014 to March 2020 with their main complaint being low back pain, and they were followed-up until the end of their treatment. We investigated associations between lumbar spondylosis, their background factors, and characteristics of the lesions and analyzed their treatment results.

**Results:**

Males had a higher prevalence of spina bifida occulta (SBO) (p = 0.0026), more lesions with bone marrow edema (p = 0.0097), and more lesions in the L5 vertebrae (p = 0.021) than females. The popular sports disciplines were baseball, soccer, and track and field in males, and volleyball, basketball, softball in females. The dropout rate, age at diagnosis, bone union rate, and treatment period did not differ between the sexes.

**Conclusion:**

Lumbar spondylolysis was more common in males than in females. SBO, bone marrow edema, and L5 lesions were more frequent in males, and sports discipline varied between the sexes.

## Introduction

Lumbar spondylolysis is the most common identifiable cause of low back pain in young athletes [1], and the lower back is one of the most affected fatigue fracture sites [2]. The main symptom of lumbar spondylolysis is low back pain during exercise, but many cases are asymptomatic [3]. Even when symptoms are present, they are often minor; therefore, many patients do not seek medical attention. However, if not properly treated, it can progress to spondylolisthesis and cause neurologic symptoms [4]; therefore, early detection and treatment of lumbar spondylolysis are very important.

The incidence of lumbar spondylolysis has been reported to differ between male and female athletes, with a male-to-female ratio of approximately 4:1 in Japan [5]. An observational study of patients who underwent abdominal and pelvic computed tomography (CT) scans for reasons unrelated to low back pain at a Japanese university hospital also reported a higher incidence rate in males [6]. However, it is unclear why lumbar spondylolysis affects males more often.

The cause of lumbar spondylolysis remains unknown in the first place. However, several candidate causes, including congenital and acquired factors, have been reported in the literature. Spina bifida occulta (SBO), which is a spinal anomaly due to a neural tube defect [7], has been reported to link to a higher occurrence of spondylolysis [6]. Several reports have suggested that spinal alignment such as lumbar lordosis [8] and pelvic tilt [9] are also associated with spondylolysis. Acquired factors include repetitive mechanical stress on the pars interarticularis, especially lumbar extension and rotation [3]. In a review of lumbar spondylolysis patients in Japan, the incidence of lumbar spondylolysis was higher in rugby and American football, judo, and baseball players [10]. Therefore, adolescent exercise time and sports discipline may play a role in the development of lumbar spondylolysis.

Because the incidence of lumbar spondylolysis differs between the sexes, we may be able to understand the factors that contribute to the development of lumbar spondylolysis by focusing on the differences between male and female patients. Moreover, the current prevention and treatment methods for lumbar spondylolysis in both males and females are the same [3]. However, providing prevention and treatment according to the individual may lower the incidence rate and enable prompt return to practice. Therefore, in this study, we retrospectively analyzed the background factors, characteristics of the lesions, and treatment results of patients with lumbar spondylolysis to determine the differences between males and females.

## Patients and method

Patients of high school age or younger who visited our institution with the chief complaint of low back pain and were diagnosed with lumbar spondylolysis from April 2014 to March 2020 were analyzed. Bilateral spondylolysis in one vertebral arch was counted as two lesions, lesions in multiple vertebrae were counted separately, and recurrent cases after bone union were counted as separate cases. Patients were divided into two groups, males and females. Patients diagnosed with lumbar spondylolysis by both magnetic resonance imaging (MRI) and CT imaging were included in this study. Patients who did not follow the treatment protocol or were lost to follow-up were excluded. The dropout rate was compared between the two groups. The age at diagnosis, presence of SBO, and sports discipline in each patient were investigated. In addition, the presence of bone marrow edema, level of the affected vertebra, pathological stage, rate of bone union, and treatment period were analyzed for each lesion and compared between the two groups.

Lumbar spondylolysis was defined as the presence of bone marrow edema in the pars interarticularis on MRI short tau inversion recovery (STIR) sequence, or the presence of a fracture line between the articular processes on CT. The treatment protocol was exercise prohibition and wearing a semi-rigid lumbosacral brace until the disappearance of bone marrow edema on MRI. In addition, physiotherapy was performed once a week from the beginning of the treatment until the patients returned to sports activities. The dropout rate was defined as the proportion of patients excluded due to lost to follow-up among those who met the inclusion criteria. SBO was defined as the presence of a bony defect in one of the vertebral arches of the lumbar or sacral vertebrae (S1, S2) on plain radiography, CT, or MRI. The pathological stages of the lesion were determined by axial slice classification using CT and MRI (as per Sairyo et al.) [11]. Bone union was defined as both the disappearance of bone marrow edema on MRI and bone union on CT. The treatment period was defined as the number of days from the date of diagnosis to the date of bone union. Pseudarthrosis lesions were excluded from the calculation of the rate of bone union, and lesions that achieved bone union were analyzed during the treatment period.

P-values for differences between the two groups were calculated from the Wilcoxon rank sum test for continuous variables and the chi-square test for dichotomous or categorical variables. The affected vertebra was analyzed between L1–L4 vs. L5, and the pathological stage was analyzed between the pre-lysis and early stages vs. progressive stage. Statistical tests were performed using R software version 4.0.5 (R Foundation for Statistical Computing, Vienna, Austria), and the epitools package.

## Results

**Fig. 1:**
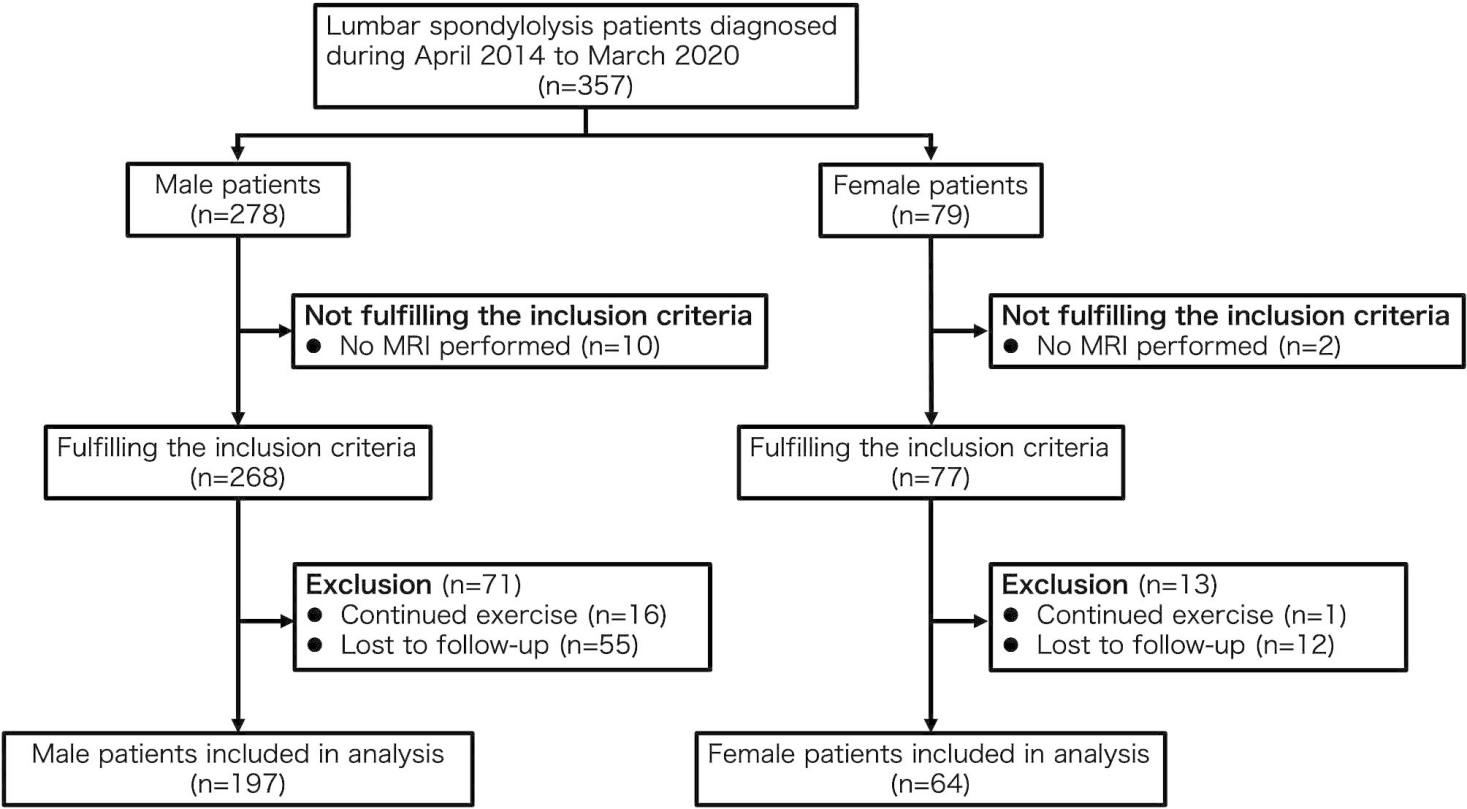
Flowchart of the study participants. Abbreviations: MRI, magnetic resonance imaging

Three hundred and fifty-seven patients were diagnosed with lumber spondylolysis during April 2014 to March 2020 in our institution. Among those patients, 345 patients, including 268 males and 77 females, were included in this study, other than 12 patients without MRI. Seventeen patients continued exercise and did not follow the treatment protocol, and 67 patients were lost to follow-up. The dropout rate was 55/268 (21%) in males and 12/77 (16%) in females (p = 0.33). As a result, a total of 261 patients with 450 lumbar spondylolysis lesions were analyzed, including 197 males with 336 lumbar spondylolysis and 64 females with 114 lumbar spondylolysis (Fig. 1). The characteristics of the cases are described in Table 1.

**Table 1:** Characteristics of cases

	Males	Females	p-value
Total cases	197	64	
Unilateral cases	73 (37%)	19 (30%)	
Bilateral cases	110 (56%)	41 (64%)	
Multiple vertebrae cases	14 (7%)	4 (6%)	
Recurrent cases	17 (9%)	2 (3%)	
Age, median (IQR)	14 (13–16)	14 (13–16)	0.66
SBO	128 (65%)	28 (44%)	0.0026

Abbreviations: IQR, interquartile range; SBO, spina bifida occulta

**Fig. 2:**
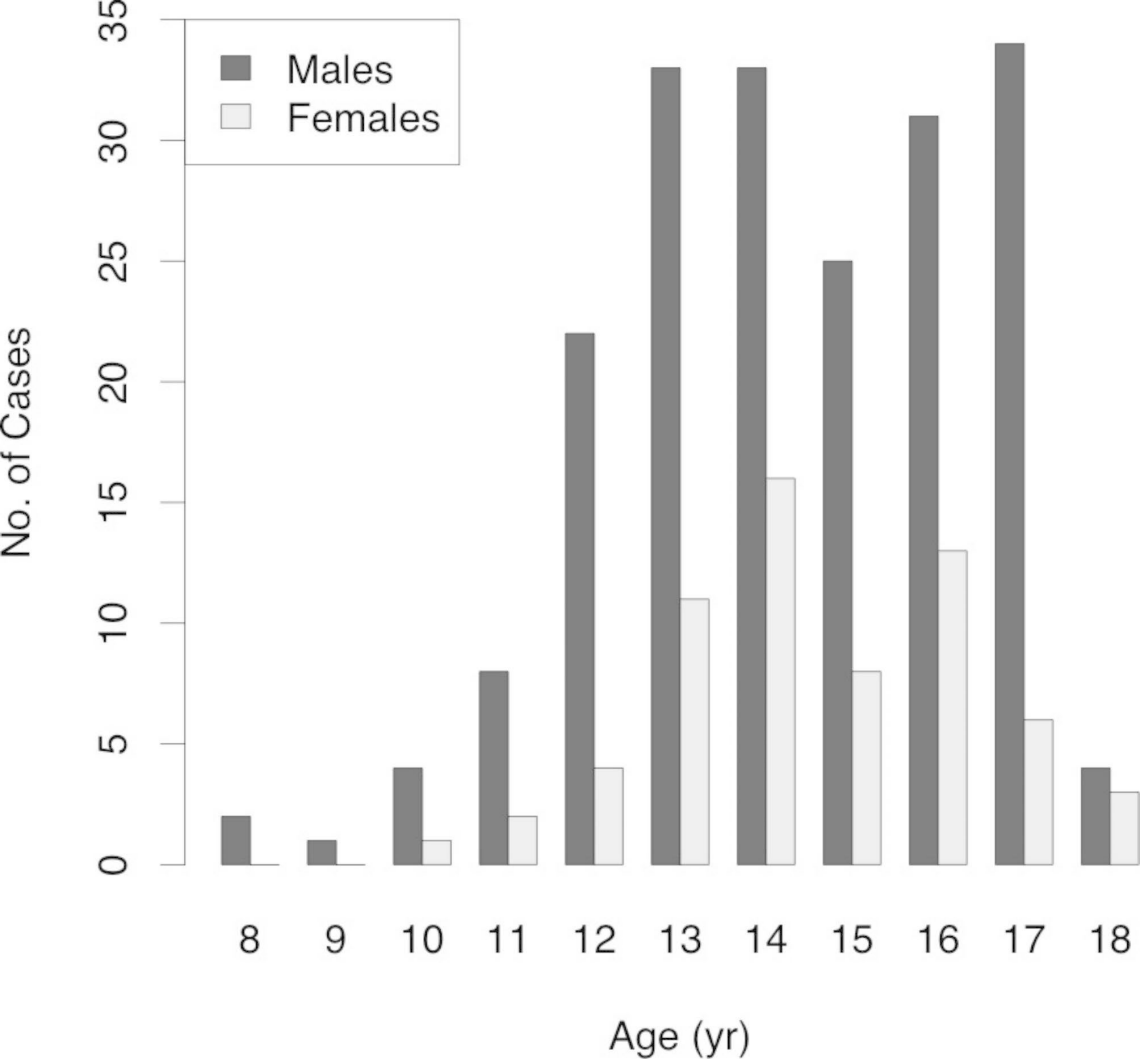
Age distribution

**Fig. 3:**
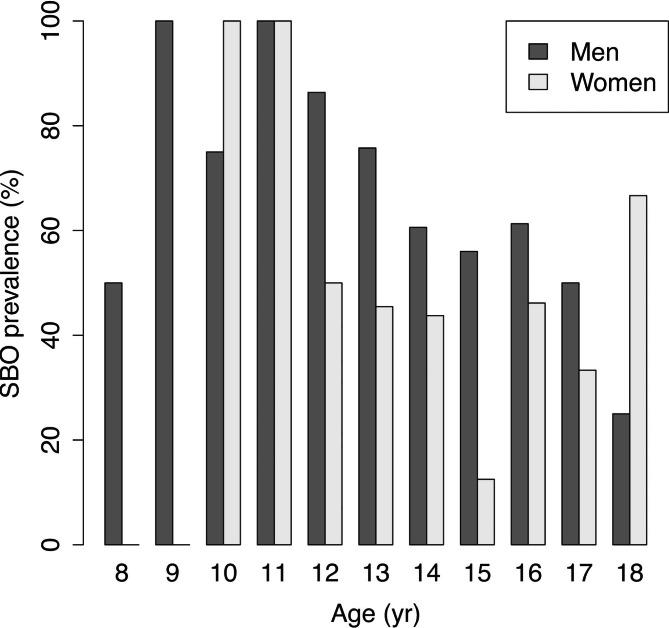
SBO prevalence by age. Abbreviations: SBO, spina bifida occulta

The median age at diagnosis was 14 years (interquartile range [IQR], 13–16 years) in both males and females, with no difference (p = 0.66, Fig. 2; Table 1). The number of patients with SBO was 128/197 (65%) in males and 28/64 (44%) in females, and the prevalence of SBO was higher in males than in females (p = 0.0026, Fig. 3; Table 1). The sport discipline differed between males and females (Table 2). Baseball, soccer, and track and field were popular among males, while volleyball, basketball, and softball were common among females.

**Table 2:** Sports disc﻿ipline of the study participants

Males		Females	
Baseball	64 (32.5%)	Volleyball	19 (29.7%)
Soccer	51 (25.9%)	Basketball	12 (18.8%)
Track and field	14 (7.1%)	Softball	6 (9.4%)
Tennis	10 (5.1%)	Soccer	5 (7.8%)
Basketball	11 (5.6%)	Tennis	5 (7.8%)
Swimming	7 (3.6%)	Track and field	5 (7.8%)
Volleyball	7 (3.6%)	None	2 (3.1%)
Table tennis	6 (3.0%)	Swimming	2 (3.1%)
Judo	5 (2.5%)	Badminton	1 (1.6%)
Softball	5 (2.5%)	Figure skating	1 (1.6%)
Karate	4 (2.0%)	Golf	1 (1.6%)
Wrestling	3 (1.5%)	Handball	1 (1.6%)
Handball	2 (1.0%)	Judo	1 (1.6%)
None	2 (1.0%)	Lifesaving	1 (1.6%)
Badminton	1 (0.5%)	Table tennis	1 (1.6%)
Cycling	1 (0.5%)	Taekwondo	1 (1.6%)
Field hockey	1 (0.5%)		
Kendo	1 (0.5%)		
Rugby	1 (0.5%)		
Taekwondo	1 (0.5%)		

There was a difference in the presence of bone marrow edema with 200/336 lesions (60%) in males and 52/114 (46%) lesions in females (p = 0.0097). The affected vertebral levels were 1 L1, 5 L2, 15 L3, 52 L4, and 263 L5 in males and 17 L3, 20 L4, and 77 L5 in females (Table 3). The prevalence of L5 lesions was higher in males than in females (78% vs. 68%, p = 0.021). The pathological stages of the lesions were 47 pre-lysis, 101 early, 52 progressive, and 136 terminal stages in males, and 16 pre-lysis, 24 early, 12 progressive, and 62 terminal stages in females (Table 3). The ratio of pre-lysis and early stages vs. progressive stage did not differ between males and females (p = 0.67).

**Table 3:** Characteristics of lesions

	Males		Females	p-value
Total lesions	336		114	
Bone marrow edema	200 (60%)		52 (46%)	0.0097
Affected vertebral levels				0.021
L1	1 (0.30%)		0 (0%)	
L2	5 (1.49%)		0 (0%)	
L3	15 (4.46%)		17 (15%)	
L4	52 (15.48%)		20 (18%)	
L5	263 (78.27%)		77 (68%)	
Pathological stage				0.67
Pre-lysis	47 (14%)		16 (14%)	
Early	101 (30%)		24 (21%)	
Progressive	52 (15%)		12 (11%)	
Bone union	145 (73%)		40 (77%)	0.52
	(*n* = 200)		(*n* = 52)	
Treatment period, median (IQR)	96 (66–129)		91.5 (71.5–111)	0.18
	(*n* = 145)		(*n* = 40)	

Abbreviations: IQR, interquartile range

**Fig. 4:**
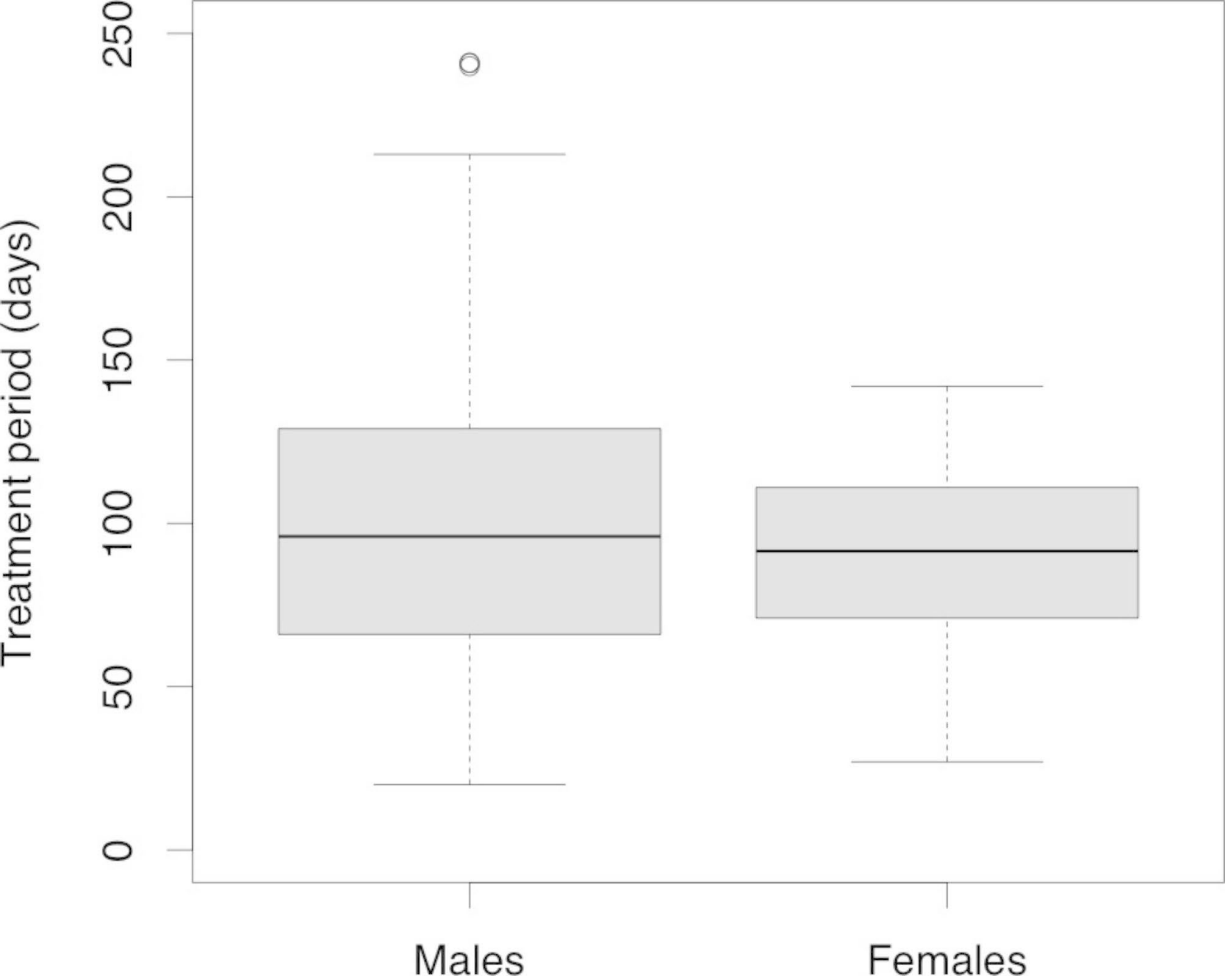
Treatment period distribution

The bone union rate was 145/200 (73%) in males and 40/52 (77%) in females, with no difference (p = 0.52). The median treatment period was 96 days (IQR, 66–129 days) in males and 91.5 days (IQR, 71.5–111 days) in females, which also showed no difference (p = 0.18, Fig. 4; Table 3).

## Discussion

Lumbar spondylolysis is the most frequent cause of low back pain in young athletes, and is more common in males than in females. The reason why males are more frequently affected by lumbar spondylolysis is unknown. Focusing on the differences between male and female patients may enable us to understand the factors that contribute to the development of lumbar spondylolysis. Furthermore, it may be possible to provide individualized prevention and treatment methods based on sex. Therefore, we analyzed 261 patients with 450 lumbar spondylolysis lesions who visited our institution to investigate the differences between male and female lumbar spondylolysis. This study found that male patients had a higher prevalence of SBO, bone marrow edema, and L5 lesions than female patients, and sports discipline differed between the sexes. The difference in the prevalence of SBO and sports discipline may be important factors related to the development of lumbar spondylolysis, while the difference in the presence of bone marrow edema and the spinal level of the affected vertebra may be useful when examining a patient with suspected lumbar spondylolysis.

Lumbar spondylolysis is more common in athletes during the growth period. One of the reasons for this is the increase in exercise intensity during this period. In addition, muscle immatureness and stiffness are common during this period when bone growth outpaces musculotendinous growth. Although there is no significant difference in bone growth between males and females until puberty, bone metabolism in females increases in early puberty and reaches its peak at mid-puberty. In contrast, bone metabolism increases slowly after puberty in males [12]; therefore, females are expected to have a lower age at diagnosis. However, in our study, there was no significant difference between the ages at diagnosis of the males and females. The proportion of males diagnosed with lumbar spondylolysis was higher than that of females, which is consistent with previous studies [5, 6, 13]. Figure 2 shows a decrease in the number of cases of males and females aged 15 years. This is likely due to the fact that third-grade junior high school students in Japan, aged 14 to 15, often take time off from sports activities to focus on their studies for high school entrance examinations.

Figure 3 shows that males had a higher prevalence of SBO than females at most ages. Moreover, younger patients had a higher prevalence in both the males and females. SBO is a spinal anomaly associated with the increased incidence of spondylolysis [6]. The prevalence of SBO among children in the general population has been reported to be higher in males and at younger ages. Among children aged 4–15 years, 51.4% of males and 32.2% of females had SBO [7]. Considering that the patients were older in this study, aged 8–18 years, the present results indicate that the same trend is observed in cases of lumbar spondylolysis, but with a much higher prevalence. The difference in SBO prevalence between males and females with lumbar spondylolysis has never been reported before, and this study is the first to show this difference. Moreover, this study indicates that the higher prevalence of SBO in males may be responsible for the higher incidence of lumbar spondylolysis in males.

Males and females participated in different sports disciplines. A review of the incidence of lumbar spondylolysis in the Japanese population reported an incidence of 16.4% among baseball players, 12.3% among basketball players, 12.0% among track and field players, 8.7% among soccer players, and 3.8% among volleyball players [10]. Although we were unable to obtain literature on the incidence of lumbar spondylolysis in Japanese softball players, if we assume that the incidence is similar to that of baseball, a high percentage of both males and females were engaged in sports disciplines that carry a high risk of developing lumbar spondylolysis. The sports disciplines with the highest incidence of lumbar spondylolysis were rugby/American football (20.5%), judo (19.7%), and baseball (16.4%) in the Japanese population [10]. Since a higher percentage of males play these sports, the differences in sports discipline may affect the incidence of lumbar spondylolysis in males and females.

The ratio of the presence of bone marrow edema in the lesions was higher in males, which is equal to the higher ratio of terminal stage lesions in females. Although the number of cases is smaller in females, the progression of lumbar spondylolysis is more advanced. Lumbar spondylolysis has been reported to be the most common cause of low back pain without neurological symptoms in males, while in females, other diseases such as undiagnosed mechanical low back pain (UMLBP) are often involved [13]. Since lumbar spondylolysis in females may include many advanced cases, it is important to make a proper early diagnosis, especially in females.

L5 was the most affected vertebral level in both males and females, but was more prevalent in males. The angle of lumbar spine lordosis has been reported to be greater in patients with L5 spondylolysis [8]. In general, as females reach puberty, their subcutaneous fat increases and body composition changes [12]. As a result, females are reported to have a greater lumbar lordosis than males [14]. Based on these reports, females are expected to suffer more L5 spondylolysis, but our data showed the opposite result. This indicates that spinal alignment alone does not determine the vertebral level of spondylolysis. Regarding the pathological stages, there was no tendency for females to have a higher incidence of progressive-stage lesions, although females had a higher incidence of terminal-stage lesions.

The bone union rate and treatment period did not differ between males and females. However, some males required a longer treatment period of approximately 8 months (Fig. 4). Although the bone union rate and treatment period are similar for males and females, we should keep in mind that some males may require an extremely long treatment period.

The limitation of this study is that we were not able to evaluate the incidence of lumbar spondylolysis in the general population. Because there may be a difference in the time between the onset of lumbar spondylolysis and the visit to the hospital for males and females, it is desirable to evaluate the status of lumbar spondylolysis in the general population. However, the present study was conducted on patients who visited our institution because MRI evaluation was necessary. In the future, it will be necessary to conduct a prospective study that includes items related to vertebral alignment, exercise time, bone metabolism, such as bone age, bone mineral density, and serum vitamin D level, and items related to the psychological aspects of patients, such as satisfaction with treatment, so that other new findings can be obtained.

## Conclusion

This study was aimed to focus on the differences between male and female lumbar spondylolysis patients. Lumbar spondylolysis was more common in males than in females. SBO, bone marrow edema, and L5 lesions were more frequent in males, and sports discipline varied between the sexes. The dropout rate, age at diagnosis, bone union rate, and treatment period did not differ between males and females.

## Data Availability

The datasets used and/or analyzed during the current study are available from the corresponding author on reasonable request.

## Abbreviations

CT: Computed tomography
MRI: Magnetic resonance imaging
STIR: Short tau inversion recovery
SBO: Spina bifida occulta
IQR: Interquartile range
UMLBP: Undiagnosed mechanical low back pain
